# Identifying the effectiveness of face mask in a large population with a network-based fluid model

**DOI:** 10.1371/journal.pone.0324229

**Published:** 2025-06-10

**Authors:** Akshay Anand, Kourosh Shoele

**Affiliations:** Department of Mechanical Engineering, Joint College of Engineering, Florida A&M University-Florida State University, Tallahassee, Florida, United States of America; Kathmandu Institute of Applied Sciences, NEPAL

## Abstract

Face masks are important in respiratory disease control, yet their effectiveness varies widely depending on the mask material and its fit on the wearer’s face. In this study, a new semi-analytical flow network model based on the Kármán-Pohlhausen technique is introduced and utilized to efficiently assess mask performance across diverse facial features that represent the observed variations inside a large population. The reduced-order model enables the evaluation of the role of different facial geometrical features with significantly lower computational costs compared to traditional computational fluid dynamics simulations. This research reveals that the area around the nose, particularly without a nose clip, is most susceptible to peripheral leakage and high-velocity jets due to larger gaps. It is argued that subtle variations in facial features, especially the zygomatic arch, significantly influence leakage patterns, emphasizing the importance of customized mask designs. The study also elucidates the complex role of nose clips in improving sealing efficacy for tightly fitted masks and redirecting leaked flow in typical imperfect facemasks. This dual function of nose clips significantly influences overall mask performance, though the exact impact varies depending on individual facial features and mask fit. The reduced-order fluid model presented here has the potential to quantify the effectiveness of face masks for a large population and influence the design of future face masks, with a focus on minimizing or redirecting leakage jets to mitigate the dispersion of respiratory aerosols thus enhancing public health strategies for respiratory disease control.

## Introduction

Despite previous research on airborne bioaerosol transmission, the COVID-19 pandemic spurred a renewed interest in understanding the underlying physics of virus transmission. Although there has been some debate, face masks are associated with being effective in slowing the infection rate [1–3]. Airborne transmission happens when someone who is not sick breathes in microscopic aerosol particles that remain floating in the air. These particles are expelled from the mouth or nose of someone who is sick during respiratory events such as coughing, sneezing, talking, or breathing [4]. To understand how airborne transmission works, it is important to study the different stages of virus travel from the infected person to the uninfected person. This includes examining how the virus is expelled from the infected person, how it travels through the air, and how it is deposited in the respiratory tract of the uninfected person. A key component of this transmission path is the presence of a face mask. Face masks are an effective way to reduce the transmission of airborne respiratory infections [5–8]. The effectiveness of face masks is clear from the predictions for the US and New York state, obtained with certain assumptions including 70% efficacy of face masks, that an adoption rate of 70% in New York state and 80% in the USA of mask could lead to the elimination of the virus [7]. Nonetheless, the exact performance of face masks is related to how they filter out aerosol particles from the air that we breathe and how they filter viruses from the exhale of the affected person.

To create effective face masks for the general public and to reach more accurate predictions for future epidemiological models, it is essential to consider essential measurements that ensure a good fit, regardless of individual facial variations.

Airborne transmission relies on the creation and dispersion of droplets, where larger droplets (≥10μm) can be effectively captured by mask fabrics due to superior filtration efficiency [9]. When these larger droplets penetrate the mask material, they are collected through various physical principles like inertial impaction, diffusion, and electrostatic deposition [10]. In contrast, smaller aerosol particles (<10μm) can escape through gaps around the mask edges, remaining suspended in the air for hours [11,12] and significantly increasing the risk of airborne virus transmission [13]. It has been found that masks made from high filtration efficiency (FE) materials can filter a wide range of particles [14]. However, it is important to note that FE alone does not account for fit. Therefore, it is recommended that fitted filtration efficiency should be considered as a more comprehensive metric as it takes into account both material filtration and mask fit. This would help in better assessing the overall effectiveness of masks in filtering particles [15].

A mask that fits well is crucial for preventing disease transmission, as it minimizes gaps around the nose, mouth, and chin, thereby preventing droplets and aerosol particles from leaking in or out. Masks made from high-quality materials, like N95 or KN95, are better at filtering small airborne particles compared to masks made from lower-quality materials like cloth, but only if they’re worn properly. However, during previous pandemics, cloth masks were more widely adopted by the population because they were easier to find at the start of the pandemic and more comfortable for extended use. Their performance, however, is highly dependent on the material properties of the mask and their proper use. While determining the optimal materials and fiber arrangements for enhancing the virus-blocking capabilities of masks is relatively straightforward, understanding the influence of mask fit on human faces poses a more complex challenge.

Earlier research on mask fit has primarily relied on targeted experiments involving a few selected human subjects or manikins (for example [16,17], among others). Both qualitative and quantitative parameters have been employed to measure leakage, ranging from subjective assessments of aerosol detection by wearers to direct measurements of particle concentrations inside and outside the mask [18]. Computational studies have established relationships between leakage and perimeter gaps for various mask types, revealing that even slight misfits can result in substantial leakage fractions, significantly undermining the protective capacity of improperly worn face masks. Their findings revealed that for a typical three-ply surgical mask with a typical misfit ($4.2\;{\text{cm}}^{2}$), the leakage fraction could be as high as 60% [19]. This substantial leakage significantly undermines the protective capacity of improperly worn face masks. While personalized face masks created using 3D facial scans have shown promise in achieving airtight seals, particularly for surgical masks [20], the development of such customized solutions for large populations remains impractical due to cost and resource constraints.

Despite previous experimental studies suggesting that mask usage can reduce the droplet and airborne transmission of various infections [21,22], there are still very limited studies about the relationship between mask protection and the facial features of people. A study by Verma *et al*. [23] experimentally investigated the effects of different kinds of masks by visualizing the respiratory jets and observing leakage through the perimeter of the mask. All masks tested experimentally exhibited leakages from the top edges due to poor fit. O’Kelly *et al*. [24] investigated the mask fit for seven participants and reported that even slight differences in facial topology could result in substantial differences in qualitative fit. Researchers from the Center for Disease Control and Prevention (CDC) suggested that proper tucking and knotting of the mask band can help achieve a better fit and improve the protective performance of face masks [25]. However, there remains a scarcity of comprehensive efforts to offer customized recommendations to the general public, taking into account factors like gender, age, and other variations in facial structures associated with body habitus.

Other studies have shown that poorly fitted N95 masks may offer even less protection than simple surgical masks [26]. Lei *et al*. [27] employed a finite element model to understand the leakage location of an N95 respirator. They reported that most leakage occurs around the upper perimeter of the mask surface near the nose. Tang *et al*. [28] explored the airflow generated during coughing and examined how the utilization of N95 and surgical masks altered the fundamental attributes of these respiratory jets. The research yielded insights indicating that while surgical masks hinder the forward movement of cough-generated airflow, their loose fit often directs leaked air primarily toward the upper, lower, and side edges. This redirection of leaked air may potentially establish a new pathway for airborne transmission of the virus. Morris *et al*. [29] explored the influence of flow pulsatility on surgical mask efficacy during coughing. They found that multi-pulsed expiratory events resulted in increased leakage around the mask compared to single-pulsed events, with leakage flow velocity showing insensitivity to mask placement. Mittal *et al*. [30] proposed the COVID-19 airborne transmission model to account for potential factors involved in airborne transmission that also consider the protection afforded by face coverings. The model is based on the filtration properties of the fabrics and aims to predict the spread of viruses to account for the uncertain effectiveness of improperly worn face masks. From the mask’s material perspective, Aydin *et al*. [31] analyzed the effectiveness of 11 commonly employed household fabrics for crafting homemade masks in terms of their ability to obstruct large, high-velocity droplets. They employed a standard commercial medical mask as a reference point for comparison. The study suggested that most fabrics have appreciably high droplet-blocking efficiency (median values > 70%). Multiple layers of highly porous fabric, such as t-shirt cloth, can block droplets with an efficiency of around 94%, similar to medical masks. Sande *et al*. [32] compared the effectiveness of several face masks and reported that homemade masks could be almost half as effective as a surgical mask and around 50 times less efficient than an FFP2 mask. The mask’s poor fit was even more noticeable on children’s faces due to the mediocre fit on smaller faces.

Solano *et al*. [33] used a wide range of realistic virtual faces to identify the leakage location and gap opening between the face and the mask inside a large population. They distinguished faces obtained from a population-based database [34] and classified them into several distinct demographics based on height, weight, age, and gender, exploring the relationship between these demographic groups and mask leakage. This work concluded that the mask design suggested by the CDC is not always the optimal solution for all demographics. Therefore, it is essential for the public to have multiple options available to better protect themselves against airborne transmission. Later, the detailed flow dynamics of representative cases from this study were computed with a high-fidelity immersed boundary approach, and the relation between peripheral leakage of the mask and its porosity for coughing condition was identified [35,36].

**Fig 1:**
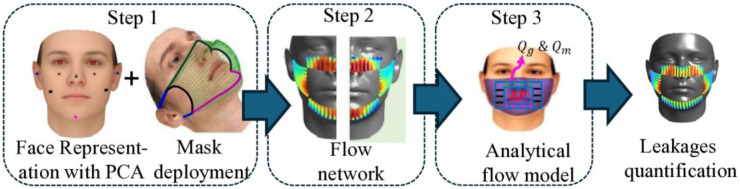
Summary of the three major steps in this research. Step 1: Face representation using PCA and mask deployment. Step 2: Development of a flow network. Step 3: Application of an analytical flow model for leakage quantification. This approach enables the quantification of both peripheral (*Q*_*g*_) and through-mask leakages (*Q*_*m*_).

Wang *et al*. [37] used a geometrically weighted principal component algorithm (PCA) to systematically morph the face and isolate the impact of different facial features on mask leakage. As illustrated in Fig 1 (first panel), facial features were correlated with the key elements of the face, allowing the classification of random faces based on six features: eyes, nose, zygomatic arch, cheeks, chin, and ears. This facial representation methodology, shown schematically in the first step of our overall approach (Fig 1), enables the systematic analysis of how facial variations affect mask fit. This research suggested that even small changes in facial features may lead to distinctive gap openings for each face, permitting non-intuitive leakage patterns at the edges of face masks.

Based on the available literature, it can be concluded that a proper "fit" is necessary to ensure the maximum effectiveness of face masks. The mask’s fit depends largely on the wearer’s facial features. However, a comprehensive understanding of the relationship between facial features, peripheral and through-mask leakage, and mask fit across a large population with diverse facial characteristics is still lacking. Many experimental studies have been conducted on limited sample sizes; making it challenging to establish statistical correlation that can be applied to larger populations [38].

Building upon the work of Wang *et al*. [37], our proposed semi-analytical flow network model overcomes this sample size limitation by enabling the systematic evaluation of mask performance across multiple virtual faces with controlled facial feature variations which would be impractical with experimental studies. Through this approach, we can establish statistically significant correlations between facial characteristics and mask leakage patterns that are generalizable to large populations.

Recently, Ni *et al*. [39] explored the use of a simplified lumped-element model to determine peripheral leakage and outward fitted filtration efficiency of face mask. They notably highlighted the significant challenge posed by peripheral leakage, demonstrating that even with an average peripheral gap of 0.65 *mm*, around 80% of exhaled airflow could escape through the mask’s periphery. Despite their work underscoring the critical importance of understanding leakage dynamics in assessing mask efficacy, they made significant assumptions to expedite mask modeling. Specifically, the study concentrated all flow resistance at the mask’s edge, neglecting internal flow patterns. Yet, our earlier detailed flow simulations [35,36] revealed the intricate flow dynamics between the face and porous mask can significantly influence both the leakage pattern and intensity around the mask’s edge. To address this limitation, we aim to develop a reduced-order model rooted in flow physics for more accurate predictions. This model essentially forms a network of interconnected channel networks, capturing the internal flow dynamics within the mask. It allows efficient assessment of mask functionality across various facial profiles by incorporating realistic, longitudinally varying peripheral gap profiles and capturing the intricate variations in gap size along the mask periphery. This enables us to examine both inward and outward protection provided by face masks in large populations. Additionally, we explore the potential impact of design enhancements, such as nose clips, on mask efficacy and provide insights into optimizing mask designs.

The flow dynamic inside the mask has many similarities to the flow dynamics of channel flow with porous boundaries. Numerous analytical and numerical solutions have been developed over the years, encompassing models for laminar channels with small [40–42] and large [43,44] suction, as well as small [45,46] and large [47,48] injection scenarios. The simplest solution to the porous channel flow can be obtained from the similarity solution used by Berman [49] where a non-linear ordinary differential equation was derived for channel flow with two porous walls and uniform injection. The equations can be solved efficiently using the perturbation method applied to a laminar flow within a channel. We utilize the same methodology here and essentially derive an analytical integral steady solution for the flow field at the varying interface between the face and mask. This is done by representing the space between the face and mask via multiple linearly interconnected channels originating from the mouth/nose (high-pressure area) and extending to the mask’s outer edge, treating the face as a solid boundary and the mask as a porous surface.

On a bigger picture, through this work, we aim to provide critical knowledge into quantifying the effectiveness of masks in the broader epidemiological context. Recent studies, such as Wang and Kavak’s work on a general epidemic model, highlight how mask design preferences can influence epidemic dynamics by emphasizing collective protection through mask usage [50]. Using the open-source individual-based epidemiology simulator JUNE, Aylett-Bullock *et al*. [51] explored how various measures, such as social interactions and masking, can influence virus spread dynamics in a large society and provide insights into effective policy interventions. Additionally, integrating artificial intelligence with mechanistic epidemiological models has shown promise in enhancing forecasting and model calibration, offering new opportunities for disease prediction and control [52]. However, in all these studies, facemask efficacy is treated as a constant, and the variability in mask effectiveness across the population is not explicitly considered.

In addition, it is known that some individuals are more likely to wear certain types of masks due to associated costs, comfort level, or availability. Without accounting for these important factors, predicting the effectiveness of various interventions and policy measures to curb the transmission of airborne diseases from any epidemiological model becomes significantly less accurate. In particular, without access to such information, the epidemiological models cannot answer important public health questions, such as: what proportion of individuals (across different demographic categories and physical settings) need to wear a mask to prevent future outbreaks or control the rate of disease spread? If there are not enough quality masks (e.g., N95 masks), how should other available mask types (e.g., surgical or cloth masks) be optimally distributed within the population [53]? Which mask attributes, based on body habitus, personal preference, or accessibility, are most effective in preventing future outbreaks [54,55]? If cloth masks or homemade masks are necessary, what guidelines would ensure their maximum effectiveness for public use? These issues are continuing challenges, and we still do not have predictive abilities if immune evasion increases the need for masks [56,57]. It also represents a long-term concern, as models that insufficiently account for the dynamics of masks hinder our preparedness for future pandemics.

The semi-analytical flow network model presented here can be utilized to address the above key shortcomings and provide quantitative measures for mask efficacy across diverse populations. Unlike traditional computational fluid dynamics simulations that are prohibitively expensive for population studies, our reduced-order model enables systematic evaluation of mask performance across multiple facial geometries at dramatically lower computational cost. This approach establishes a mechanistic relationship between facial morphology and leakage patterns, providing quantitative data that can be directly incorporated into epidemiological models. By doing so, it is possible to evaluate mask performance across diverse facial features and eventually provide valuable parameters for predictive epidemiological models to enhance their accuracy in forecasting disease spread within communities. Furthermore, lessons from the development and dissemination of infectious disease transmission models during the COVID-19 pandemic underscore the importance of learning from other pathogens to improve model reliability and applicability [58]. By combining insights from fluid dynamics and epidemiological modeling, we can better understand how mask design contributes to disease control efforts.

## Methodology

Fig 1 summarizes the methodology employed for analyzing peripheral leakage in face masks, which consists of three interconnected steps. The first step is based on our previous study, where a generative algorithm was used to create a diverse database of facial geometries [33]. This large cohort of representative faces is used to predict mask deployments using elastic energy minimization techniques (see database for faces and mask deployment simulation section for a brief discussion of the procedure, while the detailed procedure is provided in our previous publication). The outcome of this step is realistic face-mask configurations that capture variability in facial features and mask fit.

In the second step, these configurations are used to construct a detailed flow network that captures the flow structure within the face-mask interface, with particular emphasis on the formation of leakage channels (see problem setup section). This representation forms the physical basis for our mathematical model in the final step.

Finally, in the last step, we use semi-analytical fluid solutions within these channels, applying simplifying assumptions based on comparative analyses with high-fidelity simulations [35,36] (see governing equation section). This mathematical framework is used to quantitatively assess peripheral leakage under various conditions and to evaluate the combined effects of facial geometry and mask fit on flow leakage during both inhale and exhale phases.

### Database for faces and the mask deployment simulations

The Basel Face Model (BFM) [34] is used to generate a statistically representative set of facial geometries. This model utilizes principal component analysis to systematically vary facial features, allowing for a comprehensive exploration of facial diversity. To enhance control over specific facial attributes, the database is further refined by implementing the geometrically weighted principal component analysis methodology outlined by Wang *et al*. [37], which enables precise adjustments of facial characteristics prevalent in large population cohorts.

Mask deployment is simulated using an elastic energy minimization approach explained in detail in Solano *et al*. [33]. Briefly, this method accounts for the complex interactions between the deformable mask and the facial surface by minimizing the total elastic energy of the system, $\epsilon_{t}(X)$, expressed as,

$$\epsilon_{t}(X)=\epsilon_{\text{cloth}}^{s}+\epsilon_{\text{border}}^{s}+\epsilon_{\text{border}}^{b}+\epsilon_{\text{band}}^{s},$$ (1)

where $\epsilon_{\text{cloth}}^{s}$ represents the extensional elastic energy of the cloth, $\epsilon_{\text{border}}^{s}$ and $\epsilon_{\text{border}}^{b}$ denote the tension/compression and bending energies of the border strip respectively, and $\epsilon_{\text{band}}^{s}$ accounts for the tension energy in the connecting bands. Non-penetrating contact forces between the mask and face are enforced, ensuring physical realism in the simulated deployments.

The face database and mask deployment simulations described above provide the foundation for our problem setup. By applying these methods, we can generate realistic face-mask configurations that account for the variability in facial features and mask fit. These configurations will be used in the subsequent sections to analyze peripheral leakage under various conditions.

### Problem setup

**Fig 2:**
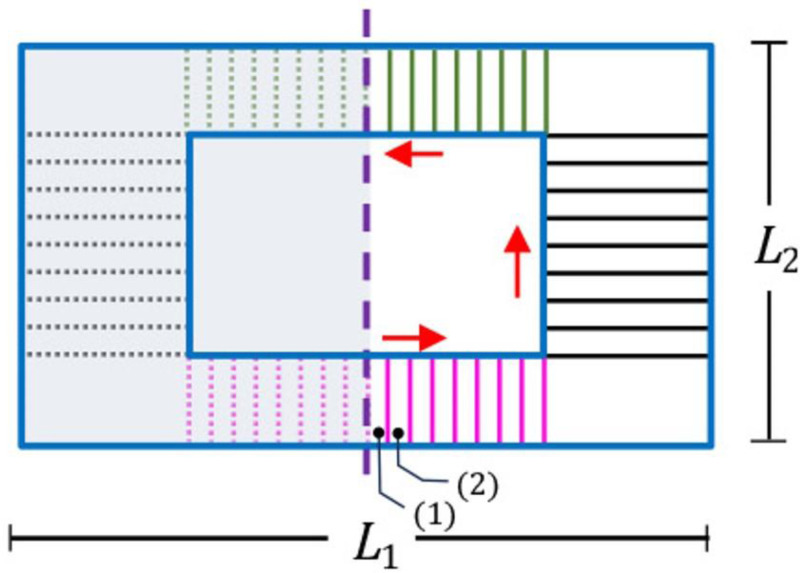
The planar view of a rectangular mask. Channels originate from the high-pressure region and terminate at the outer edge of the mask. The red arrow depicts the direction of channel numbering. Here, *L*_1_ and *L*_2_ are the sizes of the long and short edges of the mask, respectively. The shaded left side is symmetric to the right side

**Fig 3:**
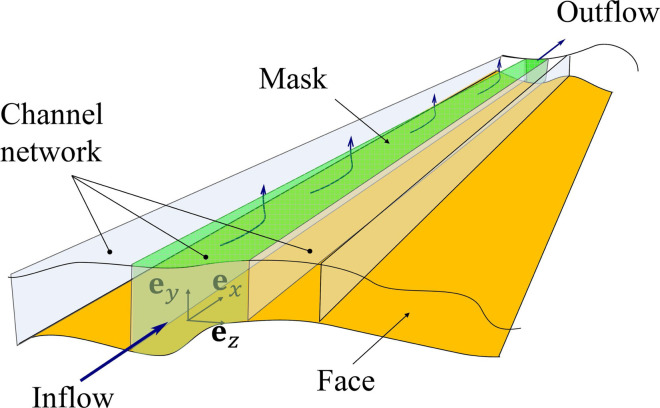
A schematic of channels defined inside the region between the face and mask.

Building upon the facial geometries and mask deployment simulations outlined in database for faces and mask deployment simulation section, we now present a detailed problem formulation for analyzing peripheral leakage in face masks. We consider a standard rectangular cloth mask measuring 9 inches by 5.4 inches, following CDC guidelines and aligning with previous research [33]. The mask geometry is illustrated in Fig 2. To accurately represent the complex flow patterns observed in high-fidelity simulations [36], the face-mask interface is represented as a network of interconnected channels, with the mask interior represented as a high-pressure region. Through comparison with high-fidelity flow simulations, we identified that the representative configuration contains a series of channels that originate from the high-pressure region in the middle of the mask and terminate on one of the edges. The final configuration contains a series of 48 interconnected channels distributed across the mask periphery, with 16 channels each in the nose and chin areas where leakage is typically more pronounced, and 8 channels on each cheek [19,35,36]. Each channel is characterized by a slowly varying height profile *K*(*x*), capturing the local gap between the face and mask. To maintain computational efficiency while preserving model fidelity, we exploit the symmetry of the face-mask system as illustrated in the non-shaded region of Fig 2 the channel numbering begins at the lower edge’s midpoint and proceeds as indicated by the red arrows. Additionally, we also show a schematic of channels defined inside the region between the face and mask in Fig 3.

Previous studies have employed various approaches to model mask leakage. Perić and Perić utilized a simplified model assuming uniform gap sizes along the mask periphery [59]. Ni *et al*. implemented lumped element model networks representing the mask system as an electrical circuit equivalent where current represents volume flow rate, voltage stands for pressure difference, and the mask fabric and peripheral gaps are modeled as parallel resistors sharing the same pressure difference across the face mask [39]. While these simplified approaches provided valuable insights with high computational efficiency, their predictions are highly dependent on calibration parameters, which could change a lot between different subjects. The model presented here addresses this shortcoming by incorporating a network of interconnected channels (see supplementary material) to more accurately represent the complex, longitudinally varying gap profiles observed in real-world scenarios. This approach allows us to identify the intricate relationship between facial features, mask fit, and leakage fluid dynamics without relying on major assumptions about fluid behavior.

While the flow motion inside the mask is complex and involves changes in all three spatial dimensions, the primary direction of the flow is parallel to the mask surface from the region next to the mouth/nose and the edges of the mask. Previous studies [23,36] have also shown this mean flow behavior. Using the channel network formulation, we can significantly reduce the computational complexity required for analyzing a wide range of scenarios. The validity of this simplification is further supported by comparisons with existing literature and experimental data, as discussed in the results section. It is important to note that while our model provides a good approximation of overall leakage behavior, it may underestimate total leakage in cases where significant flow leakage is expected or when the mask shape has a complex deployed shape. This first scenario is mostly applicable to very porous masks that do not offer a proper protection level and, therefore, are of limited practical value. To account for the complex mask shape in which there is a large space between the face and mask, one needs to use alternative techniques such as detailed three-dimensional flow simulations.

In this study, we simplify the respiratory events and only consider a steady-state process with a fixed breathing flux of 0.5 *lit*/*s* [60,61]. The primary driving force for fluid motion is the pressure difference between the region near the mouth/nose and the ambient pressure outside the mask. During exhalation, we assume uniform stagnation pressure in the mask’s interior region and flow bifurcation, with one portion directed through the channel network and the remainder passing through the porous central section of the mask. The stagnation pressure is determined inside the solution algorithm. The reverse procedure happens during inhalation.

Moreover, the tuck-in ratio of the mask is fixed at 0.5. The tuck-in ratio captures the change in the side edges due to using the connecting bands for cloth masks. It is defined as $\tau =L_{2}^{\prime }/L_{2}$, where *L*_2_ is the side edge length of the original mask and $L_{2}^{\prime }$ is the adjusted final length after deployment. This parameter plays a significant role in improving the mask’s fit on the wearer’s face, regardless of their facial topology, and the ratio of 0.5 assumed here follows the cloth mask preparation recommendation by CDC guidelines during the past COVID-19 pandemic [62].

### Governing equations

Using the geometric data from database for faces and mask deployment simulation section and flow network representation discussed in problem setup section, we can now develop a set of governing equations that can represent the flow dynamics inside variable-height channel networks that represent the space between the face and mask. Several prior works have identified analytical and semi-analytical solutions to porous channel flow. These techniques can be categorized by the flow within the porous element [40,49], size or the type of injection or suction into the porous element [63], type of boundary conditions (axial or transverse) at the porous surface [64], among others. Key inputs for the model are the variable channel height, denoted as *K*(*x*), obtained from the deployed mask-on-face geometries and the channel network as shown in Fig 2. For the following discussion, we use the flow along the ${𝐞}_{x}$ local coordinate of a sample channel with a constant width of *W* in the ${𝐞}_{z}$ and a slowly varying height of *K*(*x*) in ${𝐞}_{y}$. Furthermore, we assume that the curvature of the channel’s central line is small such that the centrifugal and secondary flow forces are negligible, and the channel can be approximated as a straight channel. The average height of the considered channel is denoted as *K*_0_ and its total length as *L*_*c*_. The general forms of continuity and momentum equations of the flow inside the channel are,

∇·𝐮=0, (2)

$$\rho \left(\partial_{t}𝐮+𝐮\cdot \nabla 𝐮\right)=-\nabla p+\mu \Delta 𝐮.$$ (3)

where $𝐮=(u,v,w{)}^{T}$ is the flow velocity and *p* is the pressure. We further assume that there is no flow along ${𝐞}_{z}$ and *w* = 0. The flow is incompressible with a constant density ρ and constant dynamic viscosity μ. The equations for *u* and *v* are nondimensionalized based on the following choices of characteristic parameters,

$$\hat{x}=\frac{x}{L_{c}},\;\hat{y}=\frac{y}{K_{0}},\;\hat{t}=\frac{t}{T},$$ (4)

$$\hat{u}=\frac{u}{U},\;\hat{v}=\frac{v}{\kappa U},\;\hat{p}=\frac{p}{P_{0}}.$$

where *T* is a characteristic timescale of the problem selected as the breathing duration, *U* is the characteristic velocity scale chosen to be the mean inflow velocity to each channel, and *P*_0_ is the characteristic pressure defined as $P_{0}=\rho U^{2}$. Here, $\kappa =K_{0}/L_{c}$ represents the average aspect ratio of the channel. With these choices of nondimensional parameters [65], the flow equations can be written as,

$$\partial_{x}u+\partial_{y}v=0,\;St\partial_{t}u+\kappa (u\partial_{x}u+v\partial_{y}u)=-\kappa \partial_{x}p+\frac{1}{Re}(\kappa^{2}\partial_{x}^{2}u+\partial_{y}^{2}u),\;\kappa [St\partial_{t}v+\kappa (u\partial_{x}v+v\partial_{y}v)]=-\frac{\partial_{y}p}{\kappa }+\frac{1}{Re}(\kappa^{2}\partial_{x}^{2}v+\partial_{y}^{2}v).$$ (5)

where *Re*, St are Reynolds and Strouhal numbers defined as,

$$Re\;=\;\frac{\rho UK_{0}}{\mu },\;\;\text{St}\;=\;\frac{K_{0}}{T\;U}.$$ (6)

Here, we drop ^^^ and use the same notation for nondimensional variables as their dimensional counterparts. In the scaling process, $T\gg K_{0}/U$ is the typical timescale of a breathing cycle and therefore St≈0. The channel height is represented through $K(x)=K_{0}\Delta h(x)$ where the nondimensional Δh(x) function represents the changes in the channel height.

#### Boundary conditions and solution procedure.

The channels are bounded by a solid face on the bottom surface at *h*_*b*_(*x*) and a porous mask surface on top at $h_{t}(x)=h_{b}(x)+\Delta h(x)$. On the upper surface, a pressure jump occurs, determined by the localized through-mask velocity at the top boundary, as described by Darcy’s law,

$$p\left(x,h_{t}(x)\right)=\frac{P_{atm}}{P_{0}}+\frac{U}{P_{0}}c_{k}v_{n}\left(x,h_{t}(x)\right).$$ (7)

where $v_{n}\left(x,h_{t}(x)\right)=v\left(x,h_{t}(x)\right)+O\left(h_{t,x}\right)$; and *c*_*k*_, is the air resistance of the fabric. At the bottom surface, we have no penetration boundary condition of $v\left(x,h_{b}(x)\right)=0$. In addition, we assume no-slip boundary conditions at both surfaces, $u\left(x,h_{b}(x)\right)=u\left(x,h_{t}(x)\right)=0$. Eq 5 can be written in compact form by rescaling *y* as $\tilde{y}=\left(h_{t}(x)-h_{b}(x)\right)y=\Delta h(x)y$. Moreover, for typical cloth facemasks, as considered here, κ is small ( κ≪1 ), allowing us to derive the leading order approximation based on κ. We drop ~ from $\tilde{y}$ hereafter to reduce equation clutter and express the the zeroth order governing equations as,

$$\partial_{x}u+\Delta h\partial_{y}v=0,$$ (8)

$$u\partial_{x}u+\Delta hv\partial_{y}u=-b\partial_{x}v(x,1)+\frac{1}{Rc}\frac{\Delta h}{\kappa }\partial_{y}^{2}u.$$ (9)

**Fig 4:**
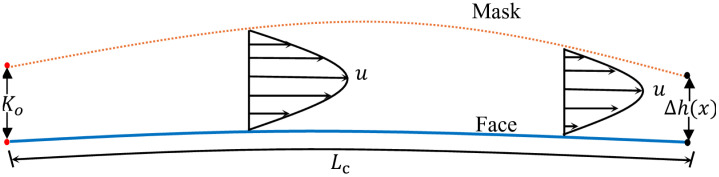
Schematics of channel flow corresponding to the points along the perimeter of the mask, *K*_*o*_ is the average height of the channel and Δh(x) is the function that varies with x; the actual local height at any point is product of *K*_*o*_ and Δh(x).

Here, $b\;=\;\frac{U}{P_{0}}c_{k}$. To solve the above equation, the Kármán-Pohlhausen momentum integral technique is used as outlined by [49,66]. In this approach, we assume a Poiseuille velocity profile for the streamwise velocity unaffected by the through-mask flow from the porous surface [49]. A schematic of the velocity profile is shown in Fig 4; the solid blue line at the bottom represents the face, and the dotted orange lines on top represent the mask, the dimensional distance between the face and the mask is $K_{0}\Delta h$. Mathematically, the streamwise velocity is written as,

u(x,y)=[1+f(x)][6η(1−η)]. (10)

where η=y/Δh(x) and from the continuity equation [64] and no penetration boundary condition at the bottom boundary with the assumption of slowly varying channel height ($\frac{d\Delta \;h}{dx}\ll \frac{df}{dx}$), the vertical velocity can be expressed as,

$$v(x,y)=-\Delta h(x)f^{\;\;\prime }\left(3\eta^{2}-2\eta^{3}\right).$$ (11)

where $f^{\prime }\equiv \frac{df}{dx}$. Furthermore, the dynamic pressure along the channel can be obtained from [64],

$$p(x)=bv(x,\eta =1)=p_{atm}-b\Delta h(x)f^{\;\;\prime }.$$ (12)

Using *u*, *v*, and *p* definitions and through the integration of the momentum equation (Eq 9) across the channel height, a second-order ordinary differential equation for *f* can be obtained. The equation is solved by imposing two boundary conditions at the inner and outer sections of the channels. At the inner section, the pressure is set to be equal to the stagnation pressure of the internal cavity, $p_{cav}$ and at the outer section, the pressure is equal to $p_{atm}\pm \frac{k_{L}}{2}{\left[1+f(1)\right]}^{2}$ where *k*_*L*_ is the minor loss due to sudden contraction at the outer section. In addition, we can calculate the unknown pressure in the mask cavity, $p_{cav}$, by setting the total flux of air leaking out of the edges and the flux of air filtering through the mask equal to the breathing flux. To eliminate the singularity associated with imposing boundary conditions based on $f^{\prime }$, we set these conditions in a different order and assume that the mean velocity at the inner section of each channel $\left({\text{U}}^{\left[\text{i}\right]}\right)$ or, equivalently, the flux at the inner section, $Q^{\left[i\right]}\;=\;U^{\left[i\right]}W\;K^{\left[i\right]}(0)$ are the unknown of the problem. We iteratively solve for the distribution of the flux between channels, $Q^{\left[1-N_{c}\right]}$, such that all channels have the same pressure at the inner section equal to dimensional $P_{cav}$ and the total flow flux entering the channel network or filtered out through the mask at the inner cavity region equals the breathing flux.

The iterative solution procedure is outlined in Algorithm 1. The algorithm begins by distributing the exhale flux between the mask cavity and the channels. It then iteratively adjusts the pressure and flux distribution across all channels until convergence is achieved. This approach effectively accommodates the inherent variability in channel heights while ensuring consistent treatment across all channels. The algorithm continues until the pressures at the inner sections of all channels converge to a common value, $P_{cav}$, and the total flux through the system matches the prescribed breathing flux.

**Algorithm 1.** Solution of the channel network for exhale cases (similar procedure with minor change is done for inhale cases). Here, ϵ is the convergence tolerance, and *a* is the flux updating step size.



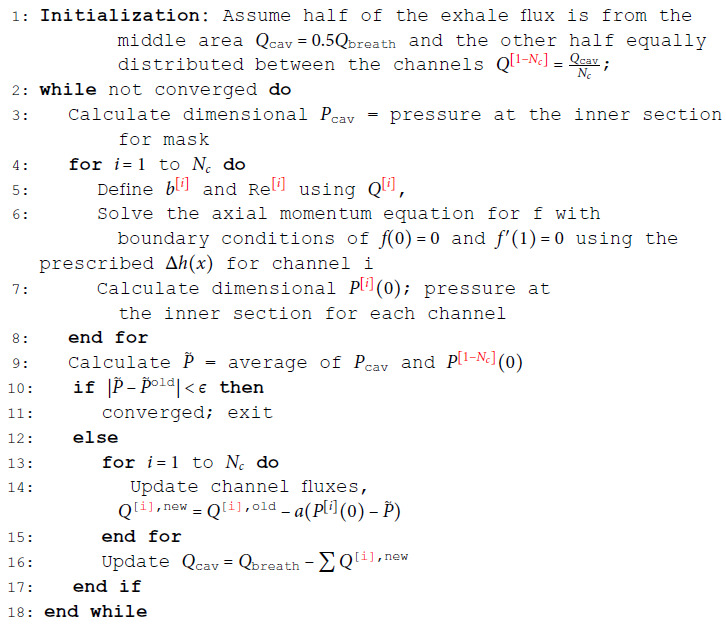



## Results and discussions

Face masks reduce virus transmission between two people through two processes: inward protection and outward protection. Inward protection prevents the wearer from inhaling virus-laden particles, while outward protection prevents the escape of virus-laden particles during the exhale phase. In the following sections, we will discuss both of these two phases. Furthermore, we will examine how different design changes of the mask, such as the addition of the nose clips, modify the peripheral leakages and the overall efficacy of the facemasks.

### Influence of mask porosity

The influence of the air resistance of the fabric *c*_*k*_ for the facial feature nose with different shape coefficients (α) is discussed next. The shape coefficient α indicates the standard deviation of the facial features within the morphed faces. The range of *c*_*k*_ is chosen from the original experimental works by Zangmeister *et al.* [15] and what has been tested in Ni *et al.* [39]. A smaller value of *c*_*k*_ means a more porous mask. From now on, we will use the symbol *Q*_*m*_ to represent the flow rate of air that permeates the mask fabric (through-flow) and use the symbol *Q*_*g*_ to denote the flow rate of air that seeps through the gaps around the mask’s edges (leakage-flow). The leakage flow per unit length of the edge is referred to as *q*_*g*_. Also, we decompose *Q*_*m*_ as $Q_{m}=Q_{m,\text{cav}}$  +  $Q_{m,\text{c}}$ where $Q_{m,\text{cav}}$ is the through-flow from the porous surface of the middle cavity region and $Q_{m,\text{c}}$ is the through-flow inside the channel network. Similarly, *Q*_*g*_ can be broken down into the summation of the leakage flow from the top (nose), bottom (chin), and sides (cheeks) of the mask, i.e. $Q_{g}=Q_{g,\text{nose}}+Q_{g,\text{chin}}+Q_{g,\text{cheeks}}$.

**Fig 5:**
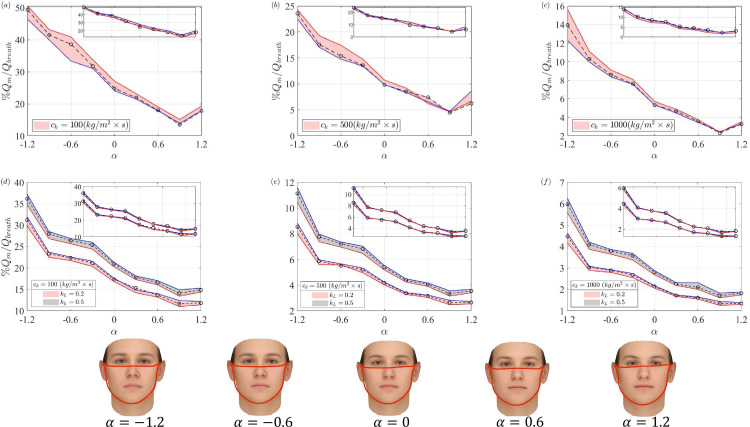
Percentage of exhale flux penetrating through the mask fabric for different values of *c*_*k*_; a–c and d–f decreasing porosity for outward and inward protection model respectively; *k*_*L*_ is head loss coefficient for inward protection model. Sensitivity analysis for the cavity size (denoted by solid red (+7.5%) and blue lines (-7.5%)); the base case is shown with dotted black lines; another sensitivity analysis for the placement of cavity regions plotted as inset plot (discussed in section influence of mask porosity) solid red lines + 5 *mm*, solid blue line -5 *mm* from the base case shown with dotted black lines.

Fig 5a–5c shows the percentage of exhaled air that penetrates through the mask fabric. As the air resistance of the mask increases (equivalently, the mask becomes less porous), more air escapes through the gaps around the edges of the mask, and less air filters through the mask fabric. This points to the first major observation that the fit of the mask is a less critical factor for more porous masks because the through-mask contribution is higher (see Fig 5a). Yet, with an increase of *c*_*k*_, a significant portion of the breath flow is redirected to peripheral leakage, and any gap from the fit or the mask during the mask deployment will contribute to a large decrease of filtered flow through the mask fabric. In fact, for masks with sufficiently high air resistance, *c*_*k*_, the overall leakage is dominated by the gaps at the edges of the mask and the space available for the flow inside the mask to reach the edges from the mid position of the mask while the porosity of the mask fabric has a secondary role. This is aligned with previous observations that even a small fractional leak area of less than 2% of the mask area can strongly deteriorate the total performance of the mask due to a sudden drop of the pressure difference between the inner mask and the outside required for pushing the flow through the mask fabrics [67]. It is also noted that while through-flow follows a similar trend for all facial features at a given *c*_*k*_, the percentage of filtered air is significantly different as different facial features yield different levels of gap opening between the mask and face and it could result in an order of magnitude modification of filter air through the mask especially for less porous masks.

Fig 5d–5f present the through-mask flow during the inhalation for two different values of loss level at the edges of the mask, *k*_*L*_. The values of *k*_*L*_ are based on published data for the minor head loss due to the sudden contraction at the edge of the mask during inhalation. The precise value of *k*_*L*_ depends significantly on the geometry of the inlet edge and the degree of rounding at the mask edge. Without loss of generality, here, two representative values of 0.2 and 0.5 are selected based on the published data for the inlet conditions of channel flow (for example [68]). With a higher value of *k*_*L*_, the percentage contribution of the *Q*_*m*_ is slightly elevated because the increased resistance of the channels in drawing air through the peripheral gap increases the pressure inside the mask and causes higher through-mask flux. Similar to the outward protection model, we notice that the percentage contribution of leakage can extend up to 50%, especially for the taller nose (α = 1.2) for less porous masks with higher *c*_*k*_. While alterations in the face shape from α=−1.2 to -0.9 may appear minor, but there is a sudden reduction in the leakage percentage. This indicates that even slight variations in facial features can lead to significant shifts in leakage distributions. The through-flow contribution consistently decreases with a higher *c*_*k*_ value, and its value drops below 10% for the largest tested *c*_*k*_ of 1000 $kg/(m^{2}\times s)$.

Given the simplified nature of the model, we performed a sensitivity analysis to quantify how much the assumed distribution of channel lengths (*L*_*c*_) around the mask’s periphery would affect the predicted leakage distribution. Three distinct configurations of channels are created along the periphery of the mask. For the base case, the center of the inner cavity region of the mask is the closest mask point to a facial point equidistant from the mouth and nose. The *y* location and its center point are further varied (with ± 5 *mm*) to account for the sensitivity with respect to the assumed inner cavity region. If the center region of the mask is shifted upward (i.e., shifted 5 *mm* from its actual location), the channels in the chin region of the face will be longer. Conversely, if it is shifted downward, the channels in the nose region will be longer, and the channels in the chin region will be shorter. All three channel configurations have almost similar behavior regarding the percentage of *Q*_*m*_ as shown in the inset plots in Fig 5a–5f. Therefore, for the rest of the study, the base case with *y* location set to zero is selected. Another sensitivity analysis is performed for the size of the inner cavity (area). In this case, we fix the cavity position as similar to the base case and vary the middle cavity area with ± 7.5% to create different configurations. For the case when the cavity area is reduced to 7.5 % it creates slightly larger *L*_*c*_ all across the periphery of the mask and in the case when the area is increased by 7.5 %, the channel lengths are smaller than the base case. In Fig 5a–5f, the sensitivity analysis for the cavity area is shown with the base case highlighted with dotted black lines. The red and blue lines are associated with ± 7.5 % changes in the cavity area. It can be seen that the trends for both inward and outward protection models remain similar, and the sensitivity of the results to the cavity size remains less than 7% at the worst cases. A detailed examination of Fig 5 reveals that the maximum deviations in through-mask flux (*Q*_*m*_) occur primarily in the outward protection model with lower porosity (*c*_*k*_ = 100 *kg*/(*m*^2^ × s), Fig 5a), where differences of 5-6% are observed at certain α values. Similar variations are seen in the corresponding inward protection model (Fig 5d). For masks with higher air resistance (*c*_*k*_ = 500 and 1000 *kg*/(*m*^2^ × s), Fig 5b–5c and 5e–5f) the sensitivity to cavity size is even smaller, typically below 4%. This relatively small variation across all tested configurations indicates that our model predictions are robust to moderate changes in cavity size assumptions, providing confidence in our baseline configuration for subsequent analyses.

**Fig 6:**
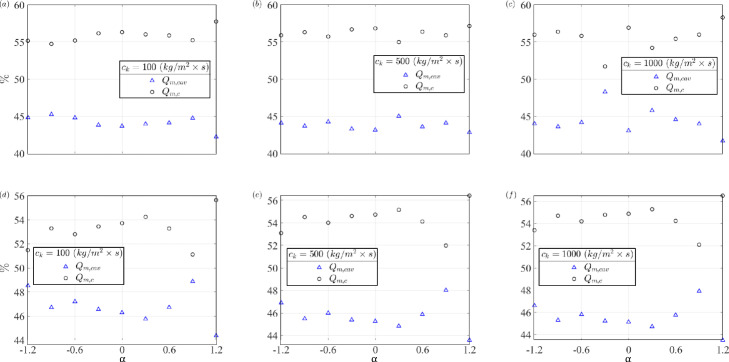
Contribution of the through-mask leakages from the cavity and channel network distributed along the periphery of the mask; a–c and d–f are decreasing porosity *c*_*k*_ = 100,500,1000 (*kg*/*m*^2^ × *s*) for outward protection (with nose clips) and inward protection model, respectively; the *k*_*L*_ for inward protection model is 0.5.

The through-mask flux *Q*_*m*_ is made up of two contributions: the through-flow from the middle cavity $Q_{m,\text{cav}}$ and the through-flow from the porous boundaries of channels $Q_{m,\text{c}}$. The relative amounts of these contributions depend on the porosity of the mask and pressure distribution in the channels. Fig 6 shows these contributions to the through-mask leakage, *Q*_*m*_. The subplot ’*a* to *c*’ and ’*d* to *f*’ represents the percentage contribution of the *Q*_*m*_ for the outward and inward protection model, respectively, assuming different *c*_*k*_ values of 100, 500, and 1000 (in $kg/(m^{2}\times s)$). The *x*–*axis* represents the shape coefficients of the facial feature nose.

For the most porous mask considered in the study with *c*_*k*_ = 100 *kg*/(*m*^2^ × s), it appears that *Q*_*m*_ contributions are roughly similar across the shape coefficient with around 55% from the channel network and 45% from the through-flow from the middle cavity region. Interestingly, this distribution remains relatively stable even for the least porous masks (for example, 1000 *kg*/(*m*^2^ × s) shown in Fig 6c and 6f). This consistency is attributed to the direct rela-tionship between channel pressure and stagnation pressure, leading to similar changes in through-mask flux for both the inner cavity and channel networks as *c*_*k*_ varies. These results suggest that while mask porosity significantly influences the relative importance of peripheral gaps and imperfect fit, the flow distribution within the mask remains relatively stable across a wide range of *c*_*k*_ values. Only at very high *c*_*k*_ values do we observe notable changes in this distribution. For the inward protection model (Fig 6d–6f, head loss coefficient = 0.5), we consistently observe that contributions from the central cavity and peripheral channel networks are of similar magnitude, further supporting the stability of this distribution across different protection scenarios.

### The distribution of peripheral leakage

The peripheral leakage around the gaps is primarily attributed to the mask’s poor fit on the wearer’s face. It is also influenced by the mask’s porosity as discussed before. The leakage distribution dictates both the direction and magnitude of the escaped flow around the mask’s edges, which is crucial for finding the role of the mask in outward protection. Here, we explore the correlation between facial features and peripheral leakage flow for four facial features – the nose, chin, cheek, and zygomatic arch. We present the results for two distinct values of $\alpha_{i}$, namely –1.2 and 1.2.

**Fig 7:**
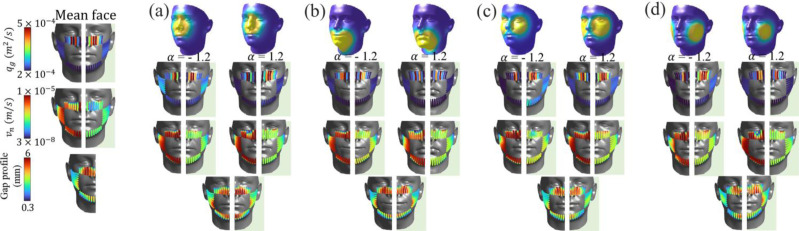
Facial shape change in row 1, peripheral leakage flux per unit width (*q*_*g*_), normal velocity ($v_{n}$), and the gap profile between the interface region between the face and the mask in rows 2, 3, and 4 respectively for (a) nose, (b) chin (c) zygomatic-arch (d) cheeks facial features for outward and inward protection model. Inward protection model results are overlayed with light green background, also, results for the mean face are shown on the extreme left.

Fig 7 illustrates the results of the flow model for each facial feature, considering both outward and inward protection scenarios. In each subplot, the results for inward protection are represented by the light green color (on the right half). The base case with α=0, indicating the mean facial shape, is included for reference, alongside α=−1.2 and α=1.2, representing maximum positive and negative deviations from the mean shape for each facial feature, respectively. The first row of Fig 7 represents the variations in face shape corresponding to different values of α. Subsequent rows provide further insights: row 2 presents the distribution of peripheral leakage per unit width, row 3 illustrates the distribution of normal velocity across the mask periphery in the channel network ($q_{m,\text{c}}$), and row 4 depicts the gap distance between the face and the mask. It’s important to note that the results only cover one-half of the face, assuming perfect symmetry (refer to problem setup section).

For all the facial features shown in Fig 7a–7d, the gap profiles reveal that the prominent openings are concentrated along the upper part of the face mask, particularly the vicinity of the nose region of the face where larger peripheral leakage occurs. The gap profiles observed around the nose regions of the mask for each facial feature exhibit variations spanning a range of 1.5 to 6 *mm*, underscoring the importance of fit quality. On the other hand, the gap profiles surrounding the chin and cheeks exhibit a narrower range, typically falling within the intervals of 0.3 to 0.5 *mm* and 0.7 to 1.2 *mm*, respectively. The consistent observation of maximum leakage concentrated around the nose region is in agreement with results obtained from previous studies that utilized optical measurement techniques [23,28] and simulations [59]. The consistency of these findings with the present work, particularly the acknowledgment that a significant portion of leakage is directed upwards from the nose region [69], enhances the validity and reliability of the current model. An additional salient observation is the substantial changes in the distribution of through-mask flux between outward and inward cases across all tested facial features. The continued presence of significant variations stresses the need to consider both outward and inward protection scenarios when assessing mask effectiveness.

### Effect of nose clip on peripheral leakage

**Fig 8:**
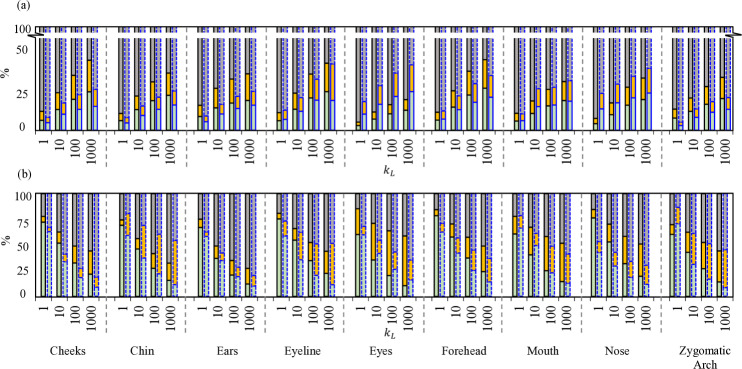
(a) Percentage contribution of peripheral leakages (*Q*_*g*_ gray), through-mask flux from the cavity ($Q_{m,\text{cav}}$ green), and the channel network ($Q_{m,\text{c}}$ orange) during exhale phase; (b) Percentage contribution of peripheral leakages (*Q*_*g*_) from the nose (green), chin (orange), and cheeks (gray). Nine different facial features are shown, and the *c*_*k*_ value is considered 500 *kg*/(*m*^2^ × s). The first bar represents the face shape α as -1.2, and next to it is negative 1.2 for each facial feature; four paired stacked bars represent *k*_*L*_ value as 10^0^, 10^1^, 10^2^, and 10^3^.

As observed in the previous section, peripheral leakage, particularly around the nose region, emerges as a significant contributor to overall mask inefficiency across various facial features. In Fig 8, we examine the impact of a wire clip with different levels of fitness on the top (nose) regions on the distribution of the outward breathing flux into peripheral leakage and through-mask flux from the mid area and channel network. The nose clip is considered to be added following the deployment of the mask and is modeled through a localized restriction at the exit section of each channel. The hydrodynamic effect of the nose clip is mathematically expressed using its associated pressure loss. The term *k*_*L*_ is varied from 1 to 1000 to represent different levels of constriction induced by the nose clip; a larger value of *k*_*L*_ represents a higher sealing level. Nine facial features are considered with two α ± 1.2 selected from the previous cases. The results are for the mask’s air resistance of *c*_*k*_ = 500 $kg/(m^{2}\times s)$. The interesting observation is that adding the nose clip does not substantially decrease the net peripheral leakage; instead, it mostly leads to redistribution of the leaked flow from the top edge to other edges of the mask.

From a design perspective, Tjolleng *et al*. investigated nose clip ergonomic design for half-face masks [70]. Their study reported a significant reduction in humidity escaping from the top (nose) region by approximately 12.6% and overall humidity by around 43% when wearing a facemask with nose clips. They discussed how such modification can enhance user comfort, addressing potential issues such as obstructed vision, particularly for wearers of glasses. While these findings are significant for user comfort, their implications from a fluid dynamics perspective are not quantified. In another study, Ortiz *et al*. conducted experiments on fourteen facemask types, affirming that outward leakage through the face seal perimeter is highly dependent on the fit around the nose and the cheeks of the manikin (test sampler), achieved by several characteristics, including the size of the mask, its fit, and the inclusion of nose clips [71]. Masks alter respiratory jet flow patterns significantly. Also, Tang *et al*. studied airflow dynamics during coughing with N95 and surgical masks, revealing diverted primary flows through leakage pathways around the mask, particularly at the top, bottom, and sides [28]. Here, we investigate the influence of nose clips on expiratory flow and jet patterns during exhalation with rectangular cloth masks. While tightly sealed nose clips effectively reduce the peripheral leakage jets and flux from the nose region, concerns persist regarding peripheral gaps around other mask edges. In fact, previous studies showed an intricate interplay between mask design elements, such as nose clips, and their implications for expiratory flow dynamics, emphasizing the necessity of a holistic approach to mask design for optimal respiratory protection.

The results of redistributed leaked flow are shown in Fig 8a where we can see that despite a gradual increase in the through-mask flux, *Q*_*m*_ by up to 20%, still the primary path for the flow is to leak from the peripheral gaps without filtering. Yet, the nose clip significantly changes the leakage flow pattern around that mask. Properly placed nose clips and a good seal around the nose lead to a substantial reduction in unfiltered leakage flux from the top edges. This redistribution of the leaked flow exhibits a strong correlation with facial features and the inward or outward phases of protection. Fig 8b shows that most of the leakage flow *Q*_*g*_ redirected to the side edges of the mask and, to a lesser extent, to the mask’s bottom edges. For instance, when examining the ’cheeks’ facial feature (the left plot in Fig 8b), for a very small *k*_*L*_ value of 1, we note that the nose region predominantly contributes to peripheral leakages due to larger gap and less resistance of airflow to escape the gap. In contrast, for the extreme scenario with *k*_*L*_ value of 1000, the cheeks (indicated by the gray color) emerge as the primary contributors to *Q*_*g*_. The redistribution of flux to the side edges is more pronounced in negative α ranges compared to positive values, attributed to modifications in the base face along the cheek feature vector and changes in mask placement during deployment. The positive α case exhibits less effective air resistance of the side channels near their exit sections, resulting in a higher percentage of flux escaping from this region. Similar trends are observed for other facial features, albeit with varying percentage contributions across different α values for each feature. These variations underscore the nuanced impact of even subtle alterations in facial characteristics, leading to distinct leakage patterns. Therefore, a thorough study of nose clip effects in a diverse population is warranted to anticipate the high level of variation across individuals.

**Fig 9:**
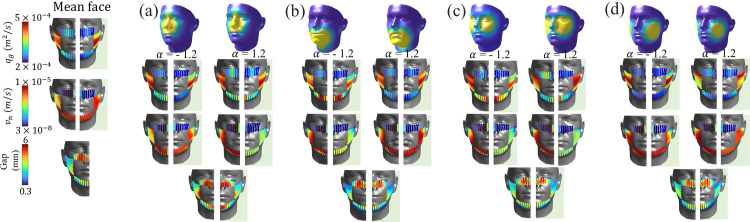
Effect of the nose clips on the peripheral leakage results of outward and inward protection model with *k*_*L*_ = 1000. Row 1: the realizations of faces when α of the corresponding feature is changed from -1.2 to +1.2. Row 2: peripheral leakage flux per unit width (*q*_*g*_). Row 3: normal velocity ($v_{n}$) and Row 4: gap distance between the interface between the face and the mask for each α for the (a) nose, (b) chin (c) zygomatic-arch (d) cheeks facial features. The results for the inward protection model are overlayed with a light green color, and mean face results for both outward and inward models are shown on the left.

Fig 9 illustrates the influence of nose clips or wires on flow dynamics during exhalation and inhalation processes. A substantial *k*_*L*_ value of 1000, representing a tight seal in the nose region, is employed to assess the impact of the nose clip. The results for inhalation cases are depicted in light green, placed next to the results from the exhalation cases. Fig 9 illustrates that, with variations in anthropometrics (facial shape), tightly placed clips have the profound ability to redirect flow away from the nose region toward the sides and bottom regions of the face, consistent with observations in Fig 8. The flow field contrasts the previously observed scenario depicted in Fig 7, where the absence of clips results in predominant leakages from the nose region. A careful observation of Fig 9 suggests a noticeable trend emerges when considering variations in the shape vector α across different facial features. Specifically, when α takes positive values; here, for example, 1.2, the peripheral leakages exhibit an increased magnitude compared to cases where α is negative. An interesting case is for the zygomatic arch Fig 9c (row 2). Here, we can notice that for this case and α value as 1.2, there is a noticeable amount of peripheral leakages from both the outward and inward models, suggesting that the zygomatic arch proves to be a critical factor influencing peripheral leakages. Even subtle modifications to the zygomatic arch can disproportionately impact the magnitude of leakages, making it a pivotal facial feature from the standpoint of face shape and mask effectiveness. As the shape vector deviates from the mean face configuration, the influence on peripheral leakages becomes more apparent, demonstrating a clear correlation between facial morphology and leakage patterns. This observation suggests the necessity for tailored mask designs that intricately consider individual facial variations to ensure optimal protection. It also exemplifies the importance of considering various variables to optimize face mask design for enhanced comfort, fit, and overall effectiveness.

### Jet velocity at the exit of mask periphery

**Fig 10:**
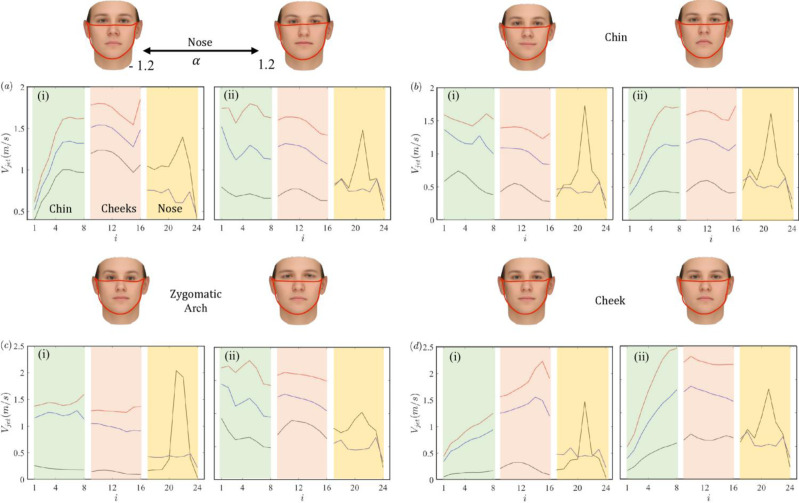
Peripheral leakage jet at the exit of mask periphery for (a) nose (b) chin (c) zygomatic-arch and (d) cheeks facial features with α equal to (i) –1.2 and (ii) 1.2 respectively. The black lines are for no clip, the blue lines are for nose clips with *k*_*L*_ = 10, and the red lines are for *k*_*L*_ = 1000. All the results are from the outward protection model. The channel numbering starts from the bottom chin position and advances to check and nose region as shown in Fig 2.

In this section, we explore the effect of the jet velocity distribution and magnitude at the exit of the periphery of the mask for four different facial features of the nose, chin, zygomatic arch, and cheeks, selected from the outward protection results presented. The faces with $\alpha_{i}=-1.2,1.2$ are selected for demonstration purposes. In Fig 10, the *i* on *x*-axis represents channel numbering, *i* can take values from 1 to 48 (*N*_*c*_ = 48); here, we show the results for one-half (*i* = 1,..,24) *N*_*c*_ based on the symmetric assumptions (discussed in problem setup section).

The escaped mean jet velocity from the channels depends on the combined effect of peripheral leakages and the effective gap between the face and the mask. In Fig 10, we show the results for cases with no clips (black lines), clip with *k*_*L*_ = 10 (blue lines), and clip with *k*_*L*_ = 1000 (red lines). It is found that when there are no nose-clips (black line), for all the facial features, the maximum jet velocity always concentrates in a confined region around the nose. This is due to the minimal aerodynamic resistance to leakage in gap between the face and mask around the nose, also noticed in Fig 7. The interface region between the face and the mask has a smaller gap around the chin and cheeks compared to the nose region. This smaller gap reduces the amount of peripheral leakages that exit via the side and bottom regions of the face. Yet, for particular cases such as nose feature and α=−1.2, there is a substantial contraction of the gap at the exit of channels and this results in large exit jet velocity in those regions even though the net flow flux is small.

A similar observation can be made for the facial features zygomatic-arch, wherein even physically insignificant changes in the faces can induce large variations in the jet velocity. Given that the distribution of viruses outside the mask is dependent on the number of escaped viruses (flow flux) and the flow velocity that disperses them in the environments, the reduction of the jet velocity around the nose can result in less upward direction dispersion of the particles around a sick individual with the mask. In return, it leads to more homogeneous particle distribution around the virus-host subject.

The maximum peak magnitude of the jet velocity from the network-based model without the nose clip ranges from 0.5 to 2.5 *m*/*s*, with significant variations across different facial features. The channel numbering system helps identify specific leakage patterns: channels *i* = 1-8 correspond to the chin region, *i* = 9-16 represent the cheeks, and *i* = 17-24 cover the nose area. Analysis of Fig 10 clearly shows that the highest jet velocities consistently appear in channels 17-24 (nose region), reaching 2.0-2.5 *m*/*s* for most facial features. This concentration of higher velocities around the nose occurs due to the minimal aerodynamic resistance and larger gaps in this region. The cheek channels (*i* = 9-16) typically exhibit moderate velocities (1.0-2.0 m/s), while chin channels (*i* = 1-8) generally show the lowest velocity magnitudes (0.5-1.5 m/s). However, this pattern can vary significantly with facial features. For example, with the zygomatic arch feature at α = -1.2, we observe a pronounced velocity spike in the nose channels, reaching nearly 2.0 m/s, while the same feature at α = 1.2 shows a more distributed profile with lower peak values.

Our predicted velocity patterns align with previous studies. Ni *et al*. [39] devised a lumped-element model to quantify the effect of peripheral leakages and they reported net jet velocities of 0.5–1.5 *m*/*s* for cloth masks during normal breathing, which corresponds well with our chin and cheek channel measurements. However, the current study shows slightly higher velocities in the nose region, likely due to our approach of incorporating variable gap profiles (1.5–6.0 *mm*) across the periphery of the face-mask configuration that account for the complex anatomical features around the nose bridge, whereas Ni *et al*. [39] employed a gap profile of 0.65 *mm* for the nominal-fit profile to maintain computational efficiency, note that nominal-fit refers to a typical mask-face fit scenario where the average gap size is consistently maintained at 0.65 *mm* across the mask periphery. This profile is used to represent a standard fit condition, providing a baseline for comparison with other variable gap profiles that account for more complex anatomical features and variations in mask fit. Another comparison is with the experimental study by Pan *et al*. [72]. They employed mannequins with and without masks along with artificially generated aerosol particles to measure outward protection efficiency as a function of particle diameter ranging from 0.5 to 5 μm. It was reported that an exhalation jet velocity is approximately 1.8 *m*/*s*, which is in the range of predictions made here. Similarly, Tang *et al*. [73] used the Schlieren optical method to visualize the airflow dynamics during nasal and mouth breathing. The study found that nasal breathing had a maximum propagation distance of 0.6 *m* with a derived velocity of 1.4 *m*/*s*, while mouth breathing had a maximum propagation distance of 0.8 *m* with a derived velocity of 1.3 *m*/*s*. The primary leakage location was identified as the nose region.

In another tangible study on spasmodic respiratory events such as coughing, Dbouk, and Drikakis [9] conducted a full-scale three-dimensional simulation to quantify velocity leakages around the periphery of a surgical mask. They reported significantly higher jet velocities of approximately 5 *m*/*s* with peripheral gaps ranging from 4 to 14 *mm*, reflecting the more forceful nature of coughing compared to normal breathing. While the absolute values differ due to the different respiratory events being considered here, the velocity profile shapes align well with those predicted by the current network model. Both studies identify peak velocities in the nose region, followed by the side edges (cheek region) and the bottom edge (chin region) of the mask. The gap size discrepancy between our model (1.5-6.0 *mm*) and Dbouk and Drikakis (4-14 *mm*) reflects the different respiratory events being modeled (breathing versus coughing) [9]. The scaling factor between these two studies (approximately 2-2.5 times) is consistent across both gap dimensions and jet velocities, providing further validation of our model’s predictive capability across different respiratory conditions. Table 1 summarizes the comparison between key observations across different modeling approaches and experimental studies focusing on respiratory flow through face masks. All studies consistently identify the nose region as the primary leakage location regardless of methodology, providing validation for our network-based model’s predictions. A more detailed comparison table including additional parameters, such as computational efficiency and relative error metrics, is available in the supplementary material.

**Table 1:** Comparison of jet velocity and peripheral gap predictions across different respiratory flow models.

Parameter	Current Model (Network-based)	Ni *et al*. (2023) (Lumped element [39])	Pan *et al*. (2021) (Experimental [72])	Tang *et al*. (2013) (Experimental [73])	Dbouk & Drikakis (2020) (Full CFD [9])
Respiratory Event	Breathing	Breathing	Breathing	Breathing	Coughing
Jet Velocity (*m*/*s*)	0.5-2.5 (varies by facial features)	0.5-1.5	1.8	1.3-1.4	5.0
Primary Leakage Location	Nose region	Nose region	Nose region	Nose region	Nose region

Next, we explore other scenarios in which clips with intermediate seals are placed (blue lines) on the nose regions. Remarkably, even in instances of sub-optimal sealing, a noticeable reduction in the peripheral jet from the nose regions is observed. This reduction, however, forces the flow to be redirected and distributed around the lateral and lower edges of the face and creates a large jet velocity in those edges. Finally, in the scenario when we considered a perfect seal (red line), we observed almost no peripheral jet from the nose region, showcasing the clip’s/seal’s action in containing the flow from escaping the mask near the top boundary. Still, what stands out is the highest jet velocity now originating from the chin and cheek regions. This observation shows the significance of clips in terms of their sealing attributes and their potential to modify flow dynamics during the exhale phase. It suggests that the optimal outward protection by the cloth mask, perhaps intermediate sealing around the nose with a similar jet velocity along all edges, could be the most effective practical path for cloth and surgical masks. The dual effects of nose clips offer an intriguing path for optimizing protective measures, particularly in scenarios where a perfect seal is challenging to achieve.

In addition to influencing peripheral leakages, the mask periphery’s jet velocity profiles highly influence respiratory aerosols’ dispersal dynamics, as mentioned above. The jet emerging from the periphery, driven by sub-optimal fits and varying facial features, disperses respiratory aerosols in lateral directions. While the peak velocities observed in our study are notably lower (around 2.5 *m*/*s*) than those generated during intense respiratory events such as coughing and sneezing (10-50 *m*/*s*) [74,75], the resultant jet profile contributes to aerosol transport. Understanding the characteristics of these aerosol dispersers is crucial for implementing effective public health measures. By designing masks that modify the jet profile, one can estimate the distances the escaped aerosols from the edges might travel. Coupled with accurate estimations of droplet transmission, this information can inform the development of efficient social distancing regulations. Such simple measures can be beneficial in mitigating the risks of respiratory disease transmission, especially in the context of early stages of epidemics, and in using surgical masks in medical facilities. Finally, the results underline the broader significance of the role of facial variation and mask design. It is important to consider both factors in the design of face masks: optimizing mask design for individual protection based on the facial feature and mask type and considering the best practical design to slow down and control the epidemic.

## Conclusions

In this study, we investigate the influence of facial shapes on peripheral leakages arising from sub-optimal face mask fits. To represent diverse population scenarios, a geometrically-weighted PCA method is employed to systematically generate faces by modifying a 3D face model based on real scans. Six major facial features are individually transmuted, creating a diverse virtual cohort with a weighting parameter (α) determining the emphasis on specific features. Utilizing the minimum elastic energy theory for realistic mask deployment and developing an analytical hydrodynamic network model of the flow inside the porous masks, we examine the efficacy of face masks during both exhalation and inhalation processes. We noted a clear relationship between mask porosity and airflow distribution both through mask fabric and around its edges. As mask porosity decreases, more airflow escapes through peripheral gaps rather than filtering through the mask fabric. This underscores the critical role of fit in mask effectiveness, particularly for less porous masks. The findings suggest that while mask fabric plays a crucial role in filtration, the fit of the mask on the wearer’s face significantly influences its overall efficacy. This highlights the importance of selecting masks with appropriate porosity levels and ensuring a proper fit to minimize leakage and maximize filtration efficiency.

Our study demonstrates a complex relationship between facial features, peripheral leakage, and jet velocity. This underscores the challenge of establishing simple relationships to predict mask effectiveness. We observed that the nose region is a major area for both peripheral leakage and jet velocity due to larger gaps and lower resistance in this area. This finding aligns with previous studies that identify the nose as a primary source of leakage in masks with sub-optimal fits [23,28,39,59,69]. The significant leakage around the nose emphasizes the critical role this facial feature plays in determining overall mask effectiveness.

Our results also suggest that subtle variations in facial features can lead to different leakage patterns, highlighting the need to account for individual anthropometric differences in mask design. For instance, individuals with pronounced zygomatic arches may experience increased leakage around the cheek area, while those with flatter noses might see more leakage around the nose. Furthermore, the correlation between leakage distribution and inward/outward protection scenarios points to the necessity of comprehensive evaluations of mask efficacy. We found that leakage patterns differ substantially between inward and outward protection models, suggesting that a mask’s effectiveness may vary depending on the direction of airflow. This emphasizes the importance of considering both scenarios when assessing mask performance and designing masks for optimal protection. Our findings on the jet velocity profiles at the mask periphery are consistent with existing literature, though slight differences were observed, likely due to the specific simulation setups and conditions of each study.

Given our observation of the nose region’s prominence in peripheral leakage, we explore potential mask design improvements, particularly focusing on nose clips. Our findings indicate that nose clips have a dual impact on both sealing efficacy and airflow redirection within the mask. While a perfect seal around all edges would effectively reduce peripheral leakage, including escaping jets from the nose, such ideal sealing is rarely achieved in practice. Instead, nose clips alter the flow dynamics within the mask, redirecting airflow towards the chin and cheeks, which can increase leakage in these areas.

The study highlights the need to consider new perspectives for refining face mask design parameters and balancing sealing efficiency with potential airflow concentration in specific facial regions. It suggests that single modifications, though seemingly effective on their own, may not be sufficient to improve the performance of realistic face masks. As an extension of this study, it would be interesting to investigate how facial morphological changes during dynamic activities, such as talking, affect mask efficacy. Specifically, examining how the movements of the mouth and lips compromise mask performance and influence peripheral leakage over time could provide deeper insights. Continued research in this area will enhance our understanding of these dynamic interactions and offer practical insights for policymakers. By integrating these findings, we can develop more physics-based approaches to establish optimal regulations and policies for future epidemics.

## Supporting information

S1 Appendix Effect of number of channels. The selection of the optimal number of channels for estimating peripheral leakages is crucial for balancing measurement accuracy and computational efficiency. We conducted a thorough analysis to determine the most effective channel configuration, considering factors such as flow complexity in different facial regions, symmetry requirements, and the trade-off between channel density and inter-channel distance. Our investigation examined configurations ranging from 36 to 56 channels, with particular attention to their impact on capturing the intricate flow dynamics around the mask periphery. A comprehensive discussion of this analysis, including detailed methodology, results, and justification for our final selection of 48 channels, is provided in the supplementary material (S1 Appendix. Effect of number of channels). This configuration was found to offer the optimal balance between accurate peripheral leakage quantification and computational resource utilization.
(PDF)

S1 Fig Parametric study for selecting an optimal number of channels. Comparison of peripheral leakage distribution across three different channel configurations (36, 48, and 56 channels) mapped onto a mean face model. The color contours represent the magnitude of peripheral leakage, with the 48-channel configuration demonstrating optimal balance between computational efficiency and accurate capture of leakage patterns, particularly in the critical nose region where leakage is most pronounced.
(PDF)

S2 Appendix Model comparison and validation. We provide a detailed comparative analysis between the current network-based model and previous computational/experimental approaches for mask flow dynamics. We show an expanded version of Table 1 with additional metrics such as computational efficiency, methodological limitations, and quantitative error estimates relative to experimental benchmarks. The comparison spans multiple respiratory conditions (breathing, coughing) and evaluates performance across key parameters including jet velocity profiles, gap measurements, and leakage distribution patterns. The comparison shows the model’s advantages in balancing computational efficiency with physical accuracy while identifying specific conditions where model predictions may require further refinement.
(PDF)

S1 Data (ZIP)
