# Supplementary material for: Identifying the effectiveness of face mask in a large population with a network-based fluid model
Source: PLoS One. 2025 Jun 10;20(6):e0324229. doi: 10.1371/journal.pone.0324229 (PMC12151480; doi:10.1371/journal.pone.0324229)
Supplement: S1 Appendix — The selection of the optimal number of channels for estimating peripheral leakages is crucial for balancing measurement accuracy and computational efficiency. We conducted a thorough analysis to determine the most effective channel configuration, considering factors such as flow complexity in different facial regions, symmetry requirements, and the trade-off between channel density and inter-channel distance. Our investigation examined configurations ranging from 36 to 56 channels, with particular attention to their impact on capturing the intricate flow dynamics around the mask periphery. A comprehensive discussion of this analysis, including detailed methodology, results, and justification for our final selection of 48 channels, is provided in the supplementary material (S1 Appendix. Effect of number of channels). This configuration was found to offer the optimal balance between accurate peripheral leakage quantification and computational resource utilization. (PDF) [file pone.0324229.s001.pdf]

Supplementary Materials: Identifying the effectiveness of face mask in a large population with a network-based fluid model

Akshay Anand and Kourosh Shoele

Department of Mechanical Engineering, Joint College of Engineering, Florida A & M University-Florida State University, Tallahassee, Florida, 32310, USA

Corresponding author: kshoele@eng.famu.fsu.edu

Effect of Number of Channels

In our study, we opted for the mean face as a representative model to illustrate the impact of the number of channels on estimating peripheral leakages. Acknowledging previous insights (Solano, 2022) (Solano, 2022) highlighting the intricacies of flow behind facemasks, particularly around the nose region, we initiated our approach with a focus on this complex area. To maintain symmetry between the nose and chin regions, an equal number of channels were allocated for both. Given the cheeks' relatively greater distance from the central cavity, a proportionally smaller number of channels were designated for this region. Initially, we distributed twelve channels each on the nose and chin regions, summing up to twenty-four channels. Additionally, six channels were allocated on each side of the cheeks, amounting to a total of thirty-six channels along the face's periphery. This configuration is designed to effectively capture the intricate flow phenomenon. However, a delicate balance exists between channel density and the pertinent distance between channels. While this arrangement ensures a comprehensive understanding of flow physics, opting for fewer channels would render the study impractical in capturing the nuanced intricacies of the flow dynamics. Therefore, to reduce the large distance between the channels, we increase the number slightly from thirty-six to forty-eight (sixteen on the nose and chin and eight on both sides of the cheek). This configuration has minimal distance between two consecutive channels, which results in better quantification of the peripheral leakages. Increasing the channels number further from forty-eight to fifty-six (twenty on both nose and chin and eight on each side of cheek) not only increases the computation time rather does not aid significantly in quantifying peripheral leakages.

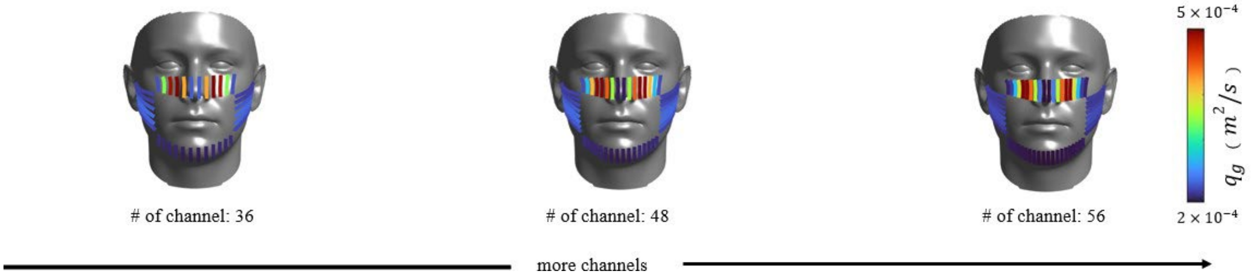

Fig. 1. *Parametric study for selecting an optimal number of channels for quantifying the flow in the interface region between the face and the mask.*

Figure 1 demonstrates the q_g for different channel configurations for the mean face. In the case of thirty-six channels, although a substantial amount of peripheral leakages from the nose regions is evident, the distance between consecutive channels exceeds the optimal range necessary for accurately quantifying these leakages. Upon increasing the channels to forty-eight, we observe that the color contours on the channels closely resemble those obtained with fifty-six channels. This

similarity indicates that both configurations effectively capture the peripheral leakages. However, with forty-eight channels, we strike a balance between computational efficiency and the ability to capture relevant features in the fluid flow. Consequently, we opt for the forty-eight channels configuration as it ensures both accuracy in peripheral leakage estimation and efficient computational resource utilization.

References

Solano T., & Shoele, K. Investigation of the role of face shape on the flow dynamics and effectiveness of face masks; Fluids, Vol. 209., p. 7(6), 2022.

Solano T., Ni, C., Mittal, R., & Shoele, K. Perimeter leakage of face masks and its effect on the mask's efficacy; Physics of fluids, p. 34(5), 2022.
