# Supplementary figures and images for: Identifying the effectiveness of face mask in a large population with a network-based fluid model
Source: PLoS One. 2025 Jun 10;20(6):e0324229. doi: 10.1371/journal.pone.0324229 (PMC12151480; doi:10.1371/journal.pone.0324229)

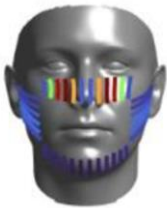

# of channel: 36

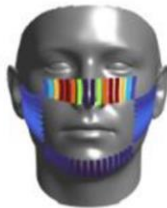

# of channel: 48

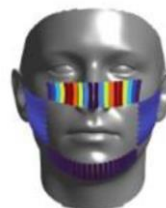

# of channel: 56

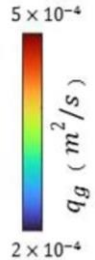

more channels

Supplement: S1 Fig — Comparison of peripheral leakage distribution across three different channel configurations (36, 48, and 56 channels) mapped onto a mean face model. The color contours represent the magnitude of peripheral leakage, with the 48-channel configuration demonstrating optimal balance between computational efficiency and accurate capture of leakage patterns, particularly in the critical nose region where leakage is most pronounced. (PDF) [file pone.0324229.s002.pdf]
