# Supplementary material for: Identifying the effectiveness of face mask in a large population with a network-based fluid model
Source: PLoS One. 2025 Jun 10;20(6):e0324229. doi: 10.1371/journal.pone.0324229 (PMC12151480; doi:10.1371/journal.pone.0324229)
Supplement: S2 Appendix — We provide a detailed comparative analysis between the current network-based model and previous computational/experimental approaches for mask flow dynamics. We show an expanded version of Table 1 with additional metrics such as computational efficiency, methodological limitations, and quantitative error estimates relative to experimental benchmarks. The comparison spans multiple respiratory conditions (breathing, coughing) and evaluates performance across key parameters including jet velocity profiles, gap measurements, and leakage distribution patterns. The comparison shows the model’s advantages in balancing computational efficiency with physical accuracy while identifying specific conditions where model predictions may require further refinement. (PDF) [file pone.0324229.s003.pdf]

Supplementary Materials: Identifying the effectiveness of face mask in a large population with a network-based fluid model

Akshay Anand and Kourosh Shoele

Department of Mechanical Engineering, Joint College of Engineering, Florida A & M University-Florida State University, Tallahassee, Florida, 32310, USA

Corresponding author: kshoele@eng.famu.fsu.edu

Model Comparison and Validation

Our network-based fluid model demonstrates good agreement with published experimental and computational studies. Predicted jet velocities (0.5-2.5 m/s) align with both computational models (Ni, 2023) which predicted 0.5-1.5 m/s and experimental measurements (Pan, 2021) which measured 1.8 m/s and other experimental work by (Tang, 2013) which reported 1.3-1.4 m/s for breathing, with relative errors of 5-15%. The variable peripheral gap measurements (1.5-6.0 mm) reflect realistic anatomical variations, more comprehensively than nominal-fit approaches in (Ni, 2023) which used 0.65 mm for their representative gap profile, while appropriately scaling compared to coughing models in (Dbouk, 2020) which used (4.0-14.0 mm). All methodologies consistently identify the nose region as the primary leakage location, validating our flow physics foundations. Our approach balances computational efficiency between simplified lumped-element models and resource-intensive 3D CFD simulations, making it particularly suitable for population-level studies examining diverse facial geometries.

Table. 1. *Comparison of key parameters across different modeling approaches and experimental studies focusing on respiratory flow through face masks. The current network-based model shows good agreement with previous computational and experimental work across multiple metrics including jet velocity, peripheral gap measurements, and leakage patterns. Differences in values reflect the varying respiratory conditions (breathing vs. coughing) and methodological approaches. Relative error metrics indicate the level of agreement between our model predictions and experimental benchmarks.*

Parameter	Current Model (Network-based)	Ni et al. (2023) (Lumped element)	Pan et al. (2021) (Experimental)	Tang et al. (2013) (Experimental)	Dbouk & Drikakis (2020) (Full CFD)
**Respiratory Event**	Breathing	Breathing	Breathing	Breathing/Coughing	Coughing
**Jet Velocity (m/s)**	0.5-2.5 (varies by facial feature)	0.5-1.5	1.8	1.3-1.4 (breathing)	5.0 (coughing)
**Peripheral Gap Range (mm)**	1.5-6.0	0.65 (constant)	Variable (not controlled)	Not specified	4.0-14.0 (coughing)
**Relative Error to Current Model**	-	~10-15%	~8-10%	~5-8%	N/A (different event)
**Primary Leakage Location**	Nose region	Nose region	Nose region	Nose region	Nose region

**Computational Efficiency**	Medium (network model)	High (simplified equations)	N/A (experimental)	N/A (experimental)	Low (full 3D CFD)

References

Dbouk T., & Drikakis, D. On respiratory droplets and face masks; Physics of Fluids, Vol. 32(6), 2020.

Ni C., Solano, T., Shoele, K., Seo, J. H., & Mittal, R. Face masks provide high outward protection despite peripheral leakage: Insights from a reduced-order model of face mask aerodynamics; Physics of Fluids, 35(6), 2023.

Pan J., Harb, C., Leng, W., & Marr, L. C Inward and outward effectiveness of cloth masks, a surgical mask, and a face shield; Aerosol Science and Technology, Vol. 55(6), pp. 718-733, 2021.

Tang J. W., Nicolle, A. D., Klettner, C. A., Pantelic, J., Wang, L., Suhaimi, A. B., & Tham, K. W. Airflow dynamics of human jets: sneezing and breathing-potential sources of infectious aerosols; PloS one. Vol. e59970, p. 8(4), 2013.
